# YOLO-CFruit: a robust object detection method for *Camellia oleifera* fruit in complex environments

**DOI:** 10.3389/fpls.2024.1389961

**Published:** 2024-08-14

**Authors:** Yuanyin Luo, Yang Liu, Haorui Wang, Haifei Chen, Kai Liao, Lijun Li

**Affiliations:** ^1^ Engineering Research Center for Forestry Equipment of Hunan Province, Central South University of Forestry and Technology, Changsha, China; ^2^ Engineering Research Center for Smart Agricultural Machinery Beidou Navigation Adaptation Technology and Equipment of Hunan Province, Hunan Automotive Engineering Vocational University, Zhuzhou, China

**Keywords:** *Camellia oleifera*, fruit detection, CBAM module, transformer, EIoU loss

## Abstract

**Introduction:**

In the field of agriculture, automated harvesting of *Camellia oleifera* fruit has become an important research area. However, accurately detecting *Camellia oleifera* fruit in a natural environment is a challenging task. The task of accurately detecting *Camellia oleifera* fruit in natural environments is complex due to factors such as shadows, which can impede the performance of traditional detection techniques, highlighting the need for more robust methods.

**Methods:**

To overcome these challenges, we propose an efficient deep learning method called YOLO-CFruit, which is specifically designed to accurately detect Camellia oleifera fruits in challenging natural environments. First, we collected images of *Camellia oleifera* fruits and created a dataset, and then used a data enhancement method to further enhance the diversity of the dataset. Our YOLO-CFruit model combines a CBAM module for identifying regions of interest in landscapes with Camellia oleifera fruit and a CSP module with Transformer for capturing global information. In addition, we improve YOLOCFruit by replacing the CIoU Loss with the EIoU Loss in the original YOLOv5.

**Results:**

By testing the training network, we find that the method performs well, achieving an average precision of 98.2%, a recall of 94.5%, an accuracy of 98%, an F1 score of 96.2, and a frame rate of 19.02 ms. The experimental results show that our method improves the average precision by 1.2% and achieves the highest accuracy and higher F1 score among all state-of-the-art networks compared to the conventional YOLOv5s network.

**Discussion:**

The robust performance of YOLO-CFruit under different real-world conditions, including different light and shading scenarios, signifies its high reliability and lays a solid foundation for the development of automated picking devices.

## 1 Introduction

*Camellia oleifera* is a unique oil tree species in China that produces a healthy to eat oil recognized by the World Food and Agriculture Organization (FAO) (Zhang and Wang, 2022). The picking season for *Camellia oleifera* fruit occurs in October each year, with a short harvesting period (Yan et al., 2020). Consequently, timely picking is crucial to ensure optimal fruit quality and quantity for maximum profitability. However, the complex and labor-intensive growth environment necessitates the use of localized picking robots to achieve high efficiency (Wang et al., 2019). These robots need to accurately identify the target crops in their natural environment to optimize the harvesting process. Therefore, timely and accurate identification of ripe *Camellia oleifera* fruit is critical for improving overall picking efficiency.

The growth environment of *Camellia oleifera* presents several technical challenges that hinder the efficiency and accuracy of machine vision systems. The primary issues include uneven lighting conditions, which can distort the color and texture features of the fruit, making it difficult for detection algorithms to differentiate between the fruit and the background. Additionally, occlusions caused by dense foliage and overlapping branches obscure the fruit from the view of picking robots, leading to a significant reduction in detection rates. These occlusions not only impede the visual access to the fruit but also create a dynamic and unpredictable environment that current machine vision systems struggle to adapt to in real-time (Zhou et al., 2022). To address these challenges, traditional image processing methods leveraging fruit color, contour, and texture features have been widely employed for detection (Chen and Wang, 2020). In scenarios where there are variations in fruit colors and backgrounds, extraction algorithms based on color and shape features have commonly been used, resulting in successful fruit segmentation (Nguyen et al., 2016; Yu et al., 2021). Despite the prevalence of traditional image processing methods that leverage fruit color, contour, and texture features, they exhibit notable limitations when the coloration of the fruit and the surrounding leaves are similar. This similarity in coloration hampers the system’s ability to accurately segment the fruit from the background, thereby limiting the overall recognition capability and leading to increased false negatives in detection (Wang et al., 2016). Consequently, some researchers have proposed a more effective detection method that combines fruit texture with color features to enhance target identification (Kurtulmus et al., 2011; Rakun et al., 2011). These studies have demonstrated that incorporating both color and texture/shape features can significantly improve fruit recognition accuracy. While traditional image processing methods offer certain benefits, they are not without their drawbacks, particularly in complex and variable scenes typical of Camellia oleifera cultivation. These methods often exhibit reduced robustness due to their sensitivity to environmental changes and the need for frequent recalibration to maintain optimal performance. The requirement for specialized calibration conditions further limits their practicality in real-world scenarios, where conditions are rarely controlled and can fluctuate widely (Gongal et al., 2015; Koirala et al., 2019).

With the rapid development of deep learning, it has emerged as a widely used tool in image processing tasks. Among the various types of deep neural networks used for visual recognition, convolutional neural networks (CNN) have shown promising outcomes (Gu et al., 2018). Currently, CNN-based object detectors can be categorized into two types: one-stage detectors and two-stage detectors. Two-stage detectors have garnered preference among researchers due to their higher accuracy and robustness. For instance, Yu et al. (Yu et al., 2019) proposed a Mask-RCNN-based model capable of detecting ripe fruit in non-structured environments, achieving an average detection precision rate of 0.957 and a recall rate of 0.954. In another study, Inkyu et al. (Sa et al., 2016) employed the Faster-RCNN model that used both RGB (red, green, and blue) and near-infrared images to detect sweet pepper. This model also demonstrated the ability to identify several other fruits, such as oranges and melons. Despite the higher accuracy and robustness of two-stage detectors, their application in the development of picking robots is significantly hindered by the substantial computational resources they require for region selection. The relatively long inference time of these detectors is a critical limitation, as it impedes real-time performance, a crucial requirement for robotic picking systems operating in dynamic and time-sensitive agricultural environments. (Fu et al., 2020) Consequently, one-stage detectors, especially the YOLO. series (Redmon et al., 2016; Redmon and Farhadi, 2017, 2018; Bochkovskiy et al., 2020; Jocher et al., 2022; Li et al., 2022; Wang et al., 2022), are becoming increasingly popular for object recognition in orchards due to their real-time detection capability and strong robustness under complex field conditions.

Tang et al. (Tang et al., 2023) proposed an improved version of the YOLOv4-tiny model for detecting *Camellia oleifera*. They utilized the k-means++ clustering algorithm to determine the bounding box prior and optimized the network structure to reduce computational complexity. The performance of this model surpassed that of both YOLOv3-tiny and YOLOv4-tiny models, achieving faster processing speed and higher average precision (AP) value. Similarly, Lu et al. (Lu et al., 2022) developed the Swim-transformer-YOLOv5 model for detecting premium grape bunches. They combined the Swim-transformer and YOLOv5 models to enhance performance. The results demonstrated that Swim-transformer-YOLOv5 outperformed Fast-er R-CNN, YOLOv3, YOLOv4, and YOLOv5 models, achieving higher average precision (AP). Wang et al. (Wang et al., 2023) used an improved YOLOv5s model to recognize and localize apples, which improved apple detection accuracy.

Vision transformers (Dosovitskiy et al., 2020), a relatively new approach in image processing, have shown promise in addressing some of the limitations of CNNs. Unlike CNNs, transformers are adept at capturing global contextual information and establishing feature dependencies through multi-head self-attention mechanisms, which can be advantageous in scenarios with occlusions and perturbations. However, the integration of transformers into practical picking robots is still in its infancy, and there are technical gaps to be bridged, such as the need for further research into how to effectively combine the strengths of transformers with the real-time requirements of robotic systems. Unlike CNNs, transformers excel in capturing global contextual information and establishing dependencies among image feature blocks using multi-head self-attentions while preserving spatial information. Several studies (Rosenfeld and Tsotsos, 2019; Huang et al., 2023) are shown that visual transformers exhibit enhanced robustness to challenges such as occlusions and perturbations compared to CNNs. Sun et al. (Sun et al., 2023) proposed the FBoT-Net model specifically for detecting small green apples. They modified the transformer layer by replacing it with a 3 × 3 convolutional layer in the last three bottleneck structures of the ResNet-50 architecture. The experimental results demonstrated impressive performance, with high average precision scores for small and large-scale apple detection on the Small Apple and Pascal VOC datasets.

To address the challenges of detecting *Camellia oleifera* fruits in natural environments, we propose an approach called YOLO-CFruit. Our approach incorporates the following strategies:

(1) Data augmentation and extension: We apply data augmentation techniques to enhance the robustness of the target detection model by augmenting the acquired image data of *Camellia oleifera* fruits.
(2) CSP bottleneck transformer (CBT) module: To enable the interaction of local and global information, we integrate the CSP structure with a transformer. This CBT module is introduced into the network backbone.
(3) CBAM integration: We incorporate the CBAM module into YOLOv5, which aids the network in recognizing regions of interest in images with large spatial coverage.
(4) EIoU loss replacement: To improve the accuracy of bounding box detection, we replace the original CIoU loss in YOLOv5 with an EIoU loss, allowing for more accurate measurement of similarity between detected bounding boxes.

In “Section 2 Materials and Methods”, we will focus on the construction of the dataset and the structural principles of the algorithm. In “Section 3 Results and Discussion”, we will verify the correctness of the structural theory analysis through experiments and evaluate the performance and discussion of our algorithm. In “Section 4 Conclusions”, we summarize the conclusions drawn from our experiments.

## 2 Materials and methods

### 2.1 *Camellia oleifera* image acquisition

The image dataset utilized in this study was obtained from a *Camellia oleifera* orchard located in Liuyang City, Hunan Province, China. The orchard follows standardized planting arrangements, with approximately 2 meters of row spacing and 1 meter of plant spacing. The *Camellia oleifera* fruit trees have a height ranging from 1 to 3 meters. During the growth period, the color of the *Camellia oleifera* fruit transitions from green to reddish brown.

**Figure 1:**
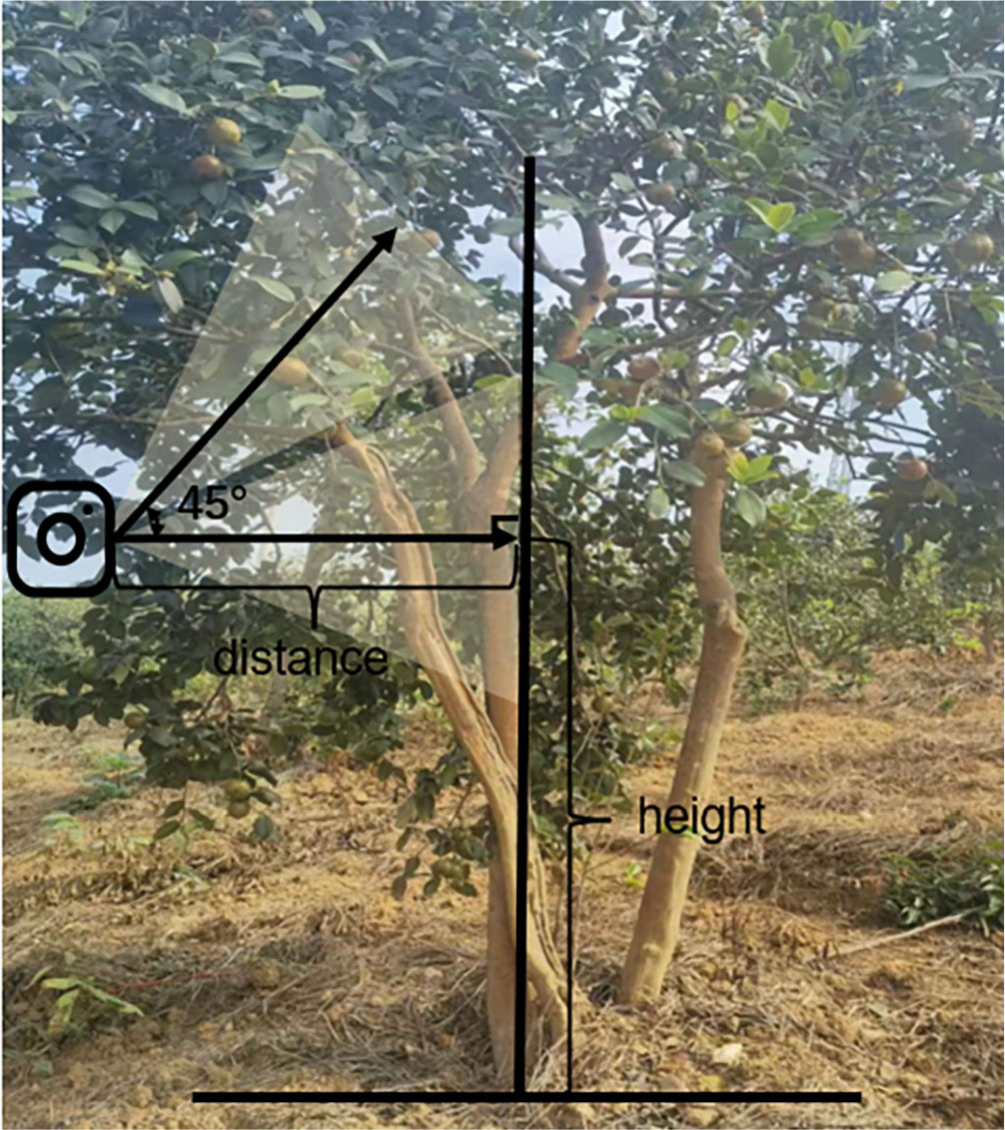
The method of image acquisition.

On October 12, 2022, image data of *Camellia oleifera* fruit were captured using iPhone 12 and saved in JPEG format with pixel resolutions of 4302 x 2268 (16:9), 3024 x 3024 (1:1).The images were captured at angles ranging from 0° to 45° with respect to the vertical line perpendicular to the tree trunk. The shooting height and distance were adjusted based on the tree’s height, ranging from 0.9 meters to 1.8 meters and 0.6 meters to 1.8 meters, respectively. Camera position in relation to tree is shown in **Figure 1**.

To enhance the recovery capability and generalization performance of the visual recognition module, the data collection process took into account the working time (from morning to evening) and weather conditions (sunny and cloudy) of the Camellia fruit robot. This approach ensured the inclusion of images captured under different lighting conditions, such as natural light, exposure variations, and backlight caused by the camera’s orientation relative to the sun’s direction. Additionally, factors like occlusion were considered.

### 2.2 Image preprocessing

**Figure 2:**
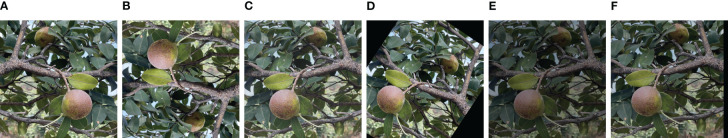
Data augmentation in the *Camellia oleifera* fruit dataset. **(A)** horizontal flip; **(B)** vertical flip; **(C)** adding noise; **(D)** random rotation; **(E)** intensity adjustment; **(F)** combination of two technologies.

The unprocessed images underwent manual annotation using the “LabelImg” software dedicated to image data annotation. Each annotated image was then stored as a txt file, containing essential information such as the object’s category, normalized central coordinates, and normalized width and height of the bounding box outlining the target. Once the entire set of original images had been meticulously annotated, the dataset has been underwent enrichment via data augmentation methodologies. These techniques include horizontal flip, vertical flip, adding noise, random rotation and intensity adjustment, or a combination of each. The original image is enhanced with different enhancement techniques, and different enhancement results are produced to augment the original data set. An example of data enhancement is shown in **Figure 2**. This augmented dataset was designed to significantly enhance the target detection model’s capacity for generalization and resilience, as outlined in reference (Liu et al., 2023).

The dataset encompasses 4,780 images of *Camellia oleifera* fruit. A stratified random sample of 3,824 images (80%) constitutes the training set, while the remaining 956 images (20%) form the validation set. This division ensures that the corresponding original and enhanced images are consistently assigned to either the training or validation set.

### 2.3 YOLOv5 model

The YOLOv5 (Jocher et al., 2022), specifically the YOLOv5s variant, stands out as an efficient target detection model with a relatively modest parameter count. This characteristic renders it particularly well-suited for real-time applications, such as those involving picking robots. The YOLOv5 model is organized into four fundamental constituents: Input, Backbone, Neck, and Head.

The Input phase involves resizing and normalization of the image to match the network’s input dimensions. The Mosaic Data Enhancement Algorithm is a variant of CutMix (CutMix) (Yun et al., 2019) that is applied to improve model training speed and network accuracy.

The backbone network comprises three central structures: the Convolution block (Conv block), the Cross Stage Partial (CSP) unit (comprising the C3 1 and C3 2 blocks), and the Spatial Pyramid Pooling-Fast (SPPF). The CSP architecture serves to amplify network depth and perceptual scope, thereby augmenting feature extraction capabilities. SPPF constitutes an upgraded iteration of the Spatial Pyramid Pooling (SPP) technique (He et al., 2015), amalgamating diverse features possessing varying resolutions to yield a more comprehensive information substrate for input into the network’s neck.

The neck networks, including FPN (Feature Pyramid Network) and PAN (Pixel Aggregation Network), fuse image features. FPN conveys semantic features from top to bottom, while PAN transmits localization features from bottom to top. The fusion of FPN and PAN enhances feature extraction in the network.

The main part of the head is three detection layers, including several components such as convolutional, pooling and fully connected layers. The detection head module uses grid-based anchor points to predict objects on feature maps from different scales of the neck.

### 2.4 Model improvements

#### 2.4.1 YOLO-CFruit network architecture

The original version of YOLOv5 adopted a pure CNN architecture, with a primary emphasis on capturing localized details. However, to account for the need for global modeling capabilities, the introduction of a transformer element becomes pertinent. Thus, a novel approach, the CSP Bottleneck Transformer module (CBT), has been devised. This module sophisticated convolution mixture and transformer structures, resulting in improved accuracy and precision in identifying *Camellia oleifera* fruit. It’s important to highlight that incorporating a vision transformer might be constrained by the quadratic computational complexity during image processing. Additionally, in cases where the network is shallow and feature mapping is extensive, an early application of the transformer layer to enforce regression boundaries could inadvertently lead to the loss of crucial contextual information, as underscored in reference (Zhang et al., 2021). In YOLOv5s, this module exclusively replaces the C3 module in layers 8 and 26.

Moreover, for enhancing the CNN’s adaptability to focus on the target and extract nuanced features, the neck network integrates the Convolutional Block Attention Module (CBAM).

**Figure 3:**
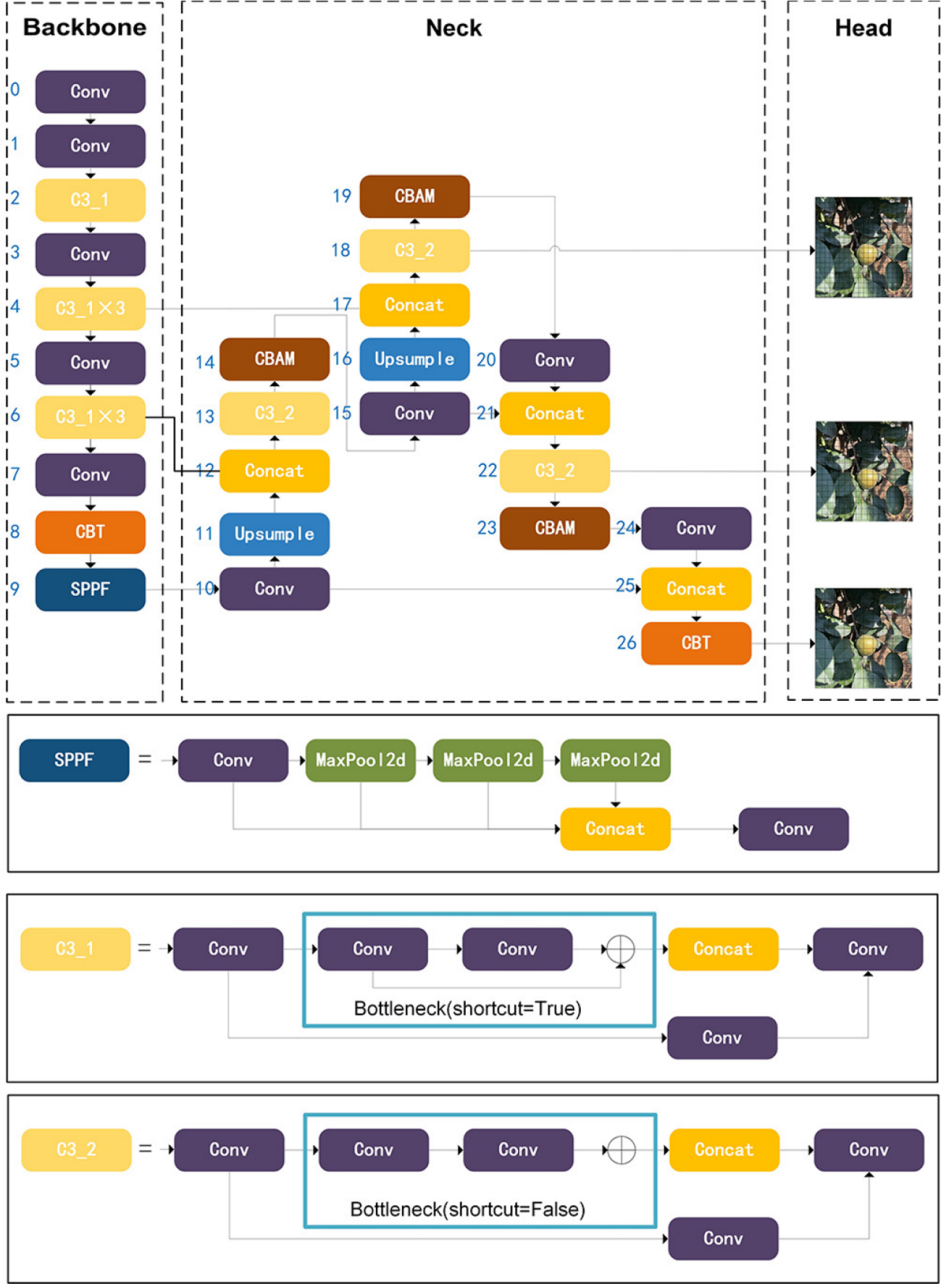
Architecture of YOLO-CFruit network.

The structural depiction of YOLO-CFruit is illustrated in **Figure 3**. Notably, this configuration boasts a low computational burden, rendering it ideally suited for the detection of *Camellia oleifera* fruit within natural environments.

#### 2.4.2 CSP Bottleneck Transformer module

**Figure 4:**
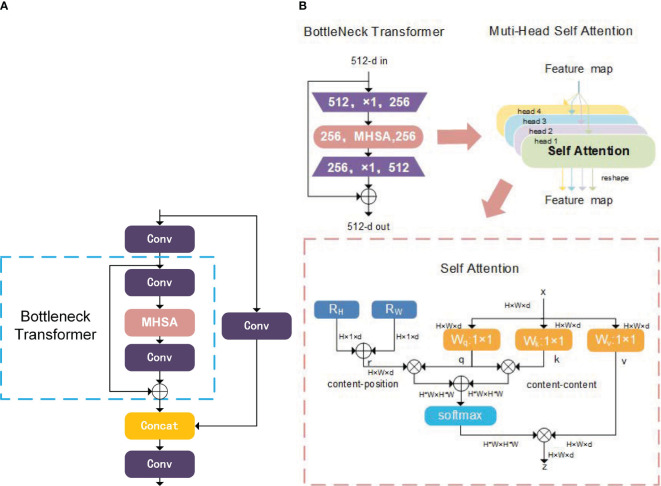
Architecture of CSP bottleneck transformer and bottleneck transformer. **(A)** Architecture of CSP Bottleneck Transformer; **(B)** Architecture of Bottleneck Transformer.

In contrast to the original C3 module, the CSP Bottleneck Transformer (CBT) demonstrates the ability to encompass both global and contextual information regarding *Camellia oleifera* fruit features. Refer to **Figure 4A** for an illustrative representation of its structure.

Traditional CNN-based models primarily aggregate local information and often struggle to capture comprehensive global insights. Conversely, Transformer-based models inherently excel at acquiring global context. The Bottleneck Transformers (BoT) block (Srinivas et al., 2021), as depicted in **Figure 4B**, harmoniously merges ResNet bottleneck components with transformer architecture, with spatial 3x3 convolutions replaced by a Multi-Head Self Attention (MHSA) layer.

Within the MHSA framework, for the self-attention pertaining to the *h*th instance, the identical input undergoes three separate 1x1 convolutions to yield the vectors *q*, *k* and *v*. Acknowledging that feature maps entail two-dimensional data, the position encodings *r* employed in self-attention mechanisms are also two-dimensional, as opposed to one-dimensional. The query $q^{h}$ , key $k^{h}$ , value $v^{h}$ , and position encoding $r^{h}$ for the hth head are shown in Equation 1.


(1) $$q^{h}=XW_{q}^{h},k^{h}=XW_{k}^{h},v^{h}=XW_{v}^{h},r^{h}=R_{H}^{h}+R_{W}^{h}$$

where *X* is the input vector, $W_{q}^{h}$ , $W_{k}^{h}$ , $W_{v}^{h}$ is the linear transformation from X to the vector *q*, *k*, *v* of *h*th head. $R_{H}^{h}$ and $R_{W}^{h}$ respectively represent relative positional information in the vertical and horizontal directions. O*^h^* represents the *h*th result of self-attention, which is computed using scaled dot-product attention. The process of calculating O*^h^* is shown in Equation 2.


(2) $$O^{h}=Softmax(q^{h}{\left(k^{h}\right)}^{T}+q^{h}\left(R_{H}^{h}+R_{W}^{h}{)}^{T}\right)v^{h}$$

#### 2.4.3 Convolutional Block Attention Module

**Figure 5:**
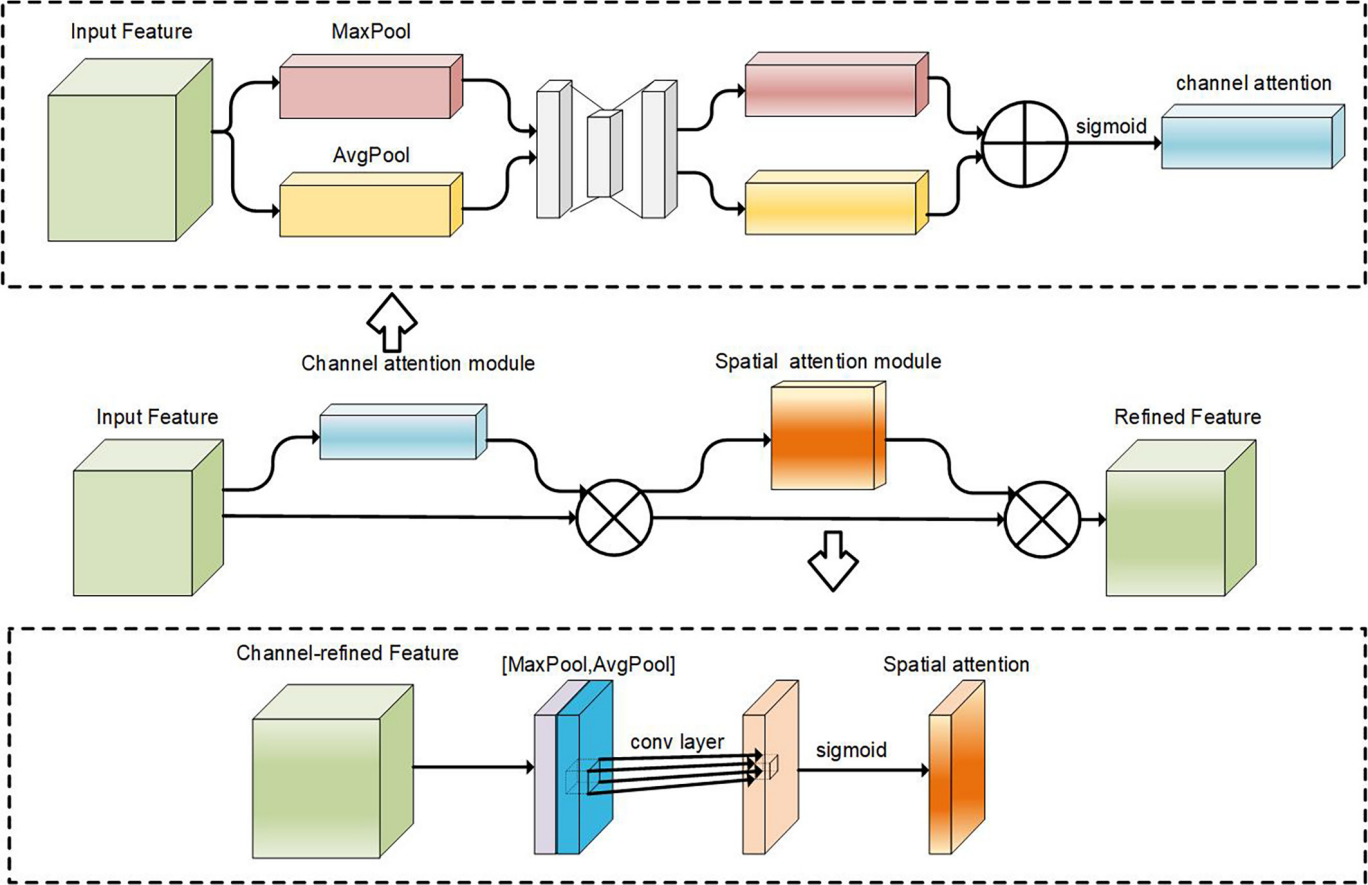
Schematic of CBAM and each attention sub-module in CBAM. ⊗ denotes element-wise multiplication.

To address the issue of foliage obscuring fruits and improve the model’s sensitivity to fruit features, this study incorporates the Convolutional Block Attention Module (CBAM) (Woo et al., 2018) within the Neck network. CBAM is an effective attention module designed for convolutional neural networks (CNNs). Its lightweight design allows seamless integration into existing CNN architectures with minimal overhead. It can be jointly trained with the base CNN, enabling end-to-end learning. The CBAM module consists of two sub-modules: the channel attention module and the spatial attention module. The process begins with the feature map traversing the channel attention module, which generates a weighted outcome. It then proceeds to the spatial attention module, further refining the weighting process. **Figure 5** provides a conceptual illustration of the CBAM module.

In the channel attention module, the input feature map F is transformed into two one-dimensional vectors using global max pooling (“MaxPool”) and global average pooling (“AvgPool”). These two vectors, with different dimensions, are passed through a multi-layer perceptron (MLP) consisting of dimensionality reduction and expansion layers. This MLP generates weight factors *W*_0_ and *W*_1_. The two one-dimensional vectors are then added element-wise, resulting in the channel attention feature map $M_{c}$ , which is activated using a sigmoid function.

The channel attention-adjusted feature map $F^{'}$ is obtained by element-wise multiplication between the original feature map *F* and the channel attention map $M_{c}$ . This modified feature map $F^{'}$ is then fed into the spatial attention module to further enhance the model’s ability to focus on relevant details.

In the spatial attention module, the channel attention-adjusted feature map $F^{'}$ undergoes global max pooling and global average pooling operations along the channel dimension, resulting in two two-dimensional vectors. These vectors are concatenated based on the channel dimension and passed through a standard convolutional layer for dimensionality reduction, resulting in a single-channel two-dimensional spatial attention map $M_{s}$ . To ensure valid values, a sigmoid activation function is applied to generate the spatial attention map $M_{s}$ . Finally, the spatial attention map $M_{s}$ and the channel attention-adjusted feature map $F^{'}$ are multiplied element-wise to obtain the final refined output $F^{\text{''}}$ . In summary, the process of CBAM is shown in Equation 3–6.


(3) $$F^{'}=M_{c}\left(F\right)⊗F$$

(4) $$F^{\text{''}}=M_{s}\left(F^{'}\right)⊗F^{'}$$

(5) $$\begin{array}{ll} M_{c}\left(F\right) & =\sigma \left(MLP\left(AvgPool\left(F\right)\right)+MLP\left(MaxPool\left(F\right)\right)\right)\\ & =\sigma \left(W_{1}\left(W_{0}\left(F_{avg}^{c}\right)\right)+W_{1}\left(W_{0}\left(F_{max}^{c}\right)\right)\right) \end{array}$$

(6) $$\begin{array}{ll} M_{s}\left(F\right) & =\sigma \left(f^{7\times 7}\left(\left[AvgPool\left(F\right);MaxPool\left(F\right)\right]\right)\right)\\ & =\sigma \left(f^{7\times 7}\left(\left[F_{avg}^{s};F_{max}^{s}\right]\right)\right) \end{array}$$

where c denotes channel attention module and s is spatial attention module. ⊗ denotes element-wise multiplication, and *σ* denotes the sigmoid function. $F_{avg}^{x}$ and $F_{max}^{x}$ represent average-pooled features and max-pooled features. Respectively, where *x* can take *c* or *s*. *f* ^(7×7)^ denotes the convolution operation where the kernel is 7 × 7.

The introduction of the Convolutional Block Attention Module (CBAM) does not alter the original spatial dimensions of the feature map. Instead, it assigns weights to each feature channel and utilizes these weights to filter out important features. This emphasis on fine-grained features allows the network to obtain improved feature mappings, leading to enhanced accuracy.

#### 2.4.4 Bounding box regression loss function

The bounding box regression loss function is a crucial method for evaluating the accuracy of model predictions and is commonly used in conjunction with Intersection over Union (IoU) (Yu et al., 2016).The IoU is calculated by dividing the intersection between the predicted box (A) and the ground truth box (B) by their union. IoU is defined as Equation 7.


(7) $$IoU=\frac{A\cap \;B}{A\cup \;B}$$

The Complete Intersection over Union Loss (CIoU Loss) algorithm (Zheng et al., 2020) introduced in YOLOv5 addresses the limitations of the traditional IoU Loss by considering the distance and aspect ratio discrepancies between the candidate bounding box and the ground truth bounding box. This provides a more comprehensive assessment of the model’s detection performance and enhances its ability to accurately locate objects. The CIoU loss is shown in **Equations 8**, **9**, where ρ is the Euclidean distance, b and *b^gt^* denote the central points of B and *B^gt^*, c is the diagonal length of the smallest enclosing box covering the two boxes, w is the width of the prediction box, h is the height of the prediction box, *w^gt^* is the width of the ground truth box, and *h^gt^* is the height of the ground truth box.


(8) $$L_{CIoU}=1-IoU+\frac{\rho^{2}\left(b,b^{gt}\right)}{c^{2}}+\alpha v\alpha =\frac{v}{\left(1-IoU\right)+v}$$

(9) $$v=\frac{4}{\pi^{2}}{\left(arctan\frac{w^{gt}}{h^{gt}}-arctan\frac{w}{h}\right)}^{2}$$

The *v* in CIoU used in YOLOv5 reflects the difference in aspect ratio rather than the difference between the width and height of the bounding box and its confidence, which can sometimes hinder the model’s effective optimization of similarity.

**Figure 6:**
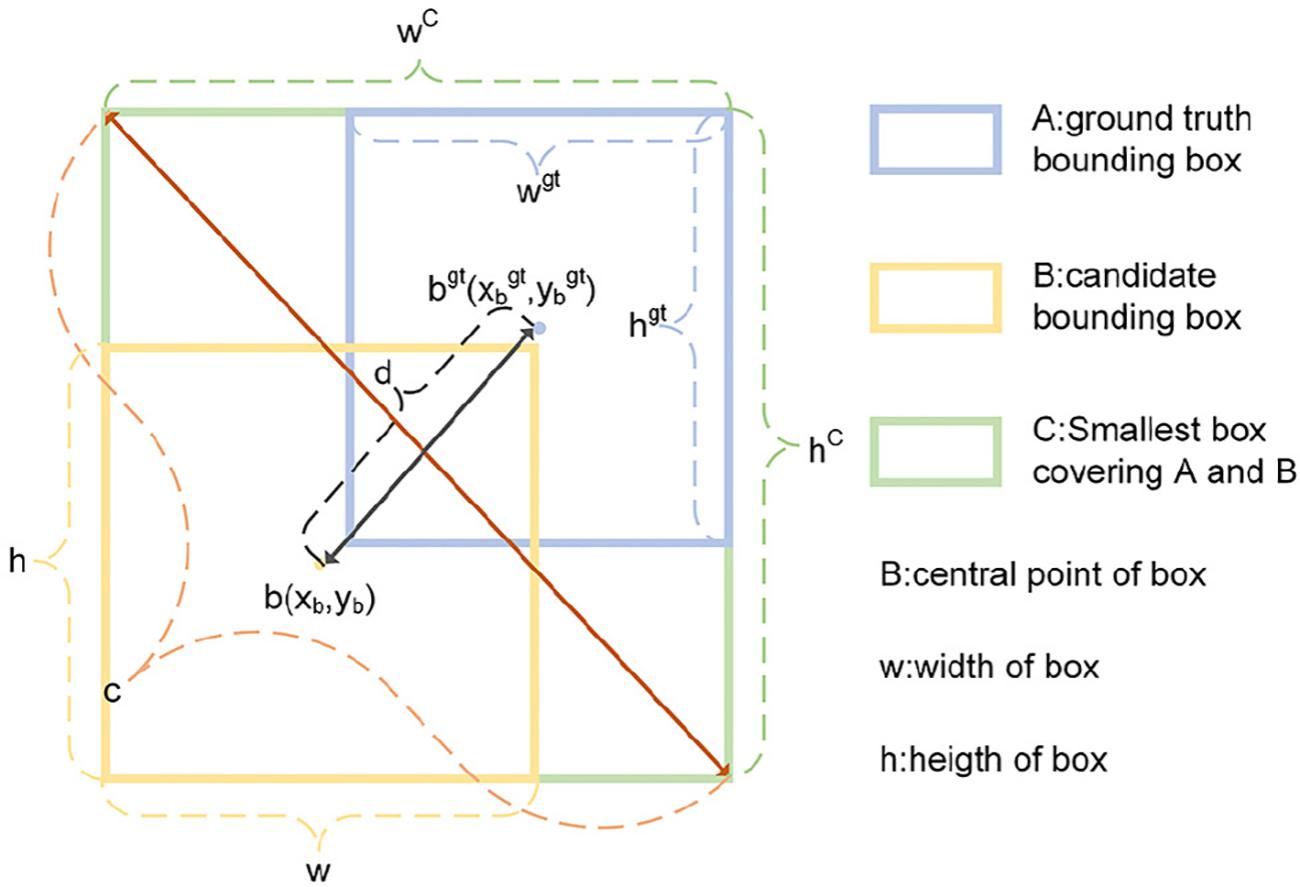
Schematic of CIoU and EIoU.

The EIoU (Zhang et al., 2022), derived from the CIoU penalty term, divides the aspect ratio impact factor into separate calculations for the target box’s length and width as well as the anchor box. The loss function is composed of three essential elements: overlap loss, center-distance loss, and width-height loss. The initial two components adopt the CIoU approach, while the width-height loss actively reduces the difference in width and height between the target box and the anchor box. This results in a more rapid convergence. EIoU is defined as Equation 10.


(10) $$L_{EIoU}=1-IoU+\frac{\rho^{2}\left(b,b^{gt}\right)}{c^{2}}+\frac{\rho^{2}\left(w,w^{gt}\right)}{w^{c}}+\frac{\rho^{2}\left(h,h^{gt}\right)}{h^{c}}$$

where $w^{c}$ and $h^{c}$ are the width and height of the minimum bounding box that covers both boxes. The schematic of CIoU and EIoU is presented in **Figure 6**.

### 2.5 Evaluation indicators of network model

The performance evaluation of *Camellia oleifera* fruit detection in this study utilized four indicators: Precision, Recall, F1 score, and Average Precision (AP). These parameters are commonly used in object detection tasks to assess the accuracy and effectiveness of the detection model. These parameters are defined as shown in Equations 11–14:


(11) $$Precision=\frac{TP}{\left(TP+FP\right)}\times 100\%$$

(12) $$Recall=\frac{TP}{\left(TP+FN\right)}\times 100\%$$

(13) $$F1=2\times Precision\cdot \frac{Recall}{Precision+Recall}$$

(14) $$AP=\int_{0}^{1}\left(Precision\cdot Recall\right)dRecall$$

where TP represents the number of true positives (i.e., positive samples predicted as positive), FN represents the number of false negatives (i.e., negative samples predicted as negative), and FP denotes the number of false positives (i.e., negative samples predicted as positive). The intersection set IoU indicates the overlap ratio between the predicted bounding box and the true bounding box. Typically, the IoU threshold is set to 0.5, where samples with an IoU greater than 0.5 are considered true positives, while those with an IoU less than 0.5 are considered false positives.

Higher values of Precision, Recall, F1 score, and AP indicate better performance in *Camellia oleifera* fruit detection. Precision reflects the accuracy of fruit recognition by the network, while Recall indicates the ability to correctly detect all instances of *Camellia oleifera* fruits. The F1 score combines Precision and Recall into a single metric, providing a balance between the two. Average Precision (AP) measures the overall detection performance across different recall levels.

### 2.6 Training platform

Training was conducted on a computer equipped with Intel Xeon W-2223 CPU processor with 128GB RAM, and NVIDIA RTX A4000 GPU. The software tools used include CUDA 11.1, CUDNN 7.6.5, OpenCV3.4.1, and Visual Studio 2017.

The detection model for *Camellia oleifera* fruit was established through the finetuning of the YOLOv5s model using the self-made *Camellia oleifera* fruit dataset and transfer learning. The YOLOv5s model was utilized to initialize the configuration parameters. YOLO-CFruit receives input images of 640 × 640 pixels, 32 batch size, 0.01 learning rate and 150 epochs for training.

## 3 Results and discussion

### 3.1 Ablation experiment with different modifications

The proposed method aims to improve the accuracy of object detection in the YOLO-CFruit model by integrating the CBT module, CBAM attention module to improve the network structure of YOLOv5s and improving the loss function. To evaluate the effectiveness of this method, ablation experiments were conducted by removing each of the improved modules one at a time and training the model to measure the impact of the modifications. The goal was to identify specific substructures of the model and optimize them for better performance. To ensure the validity of the experiments, the model was trained using consistent hyperparameters and operating environment.

**Table 1** shows the results of the ablation experiments, with mean average precision and F1 score used as the evaluation metrics. The modifications made to different parts of the model had a positive impact on its accuracy. Notably, compared to the original YOLOv5, the EIoU module has the most significant impact. The EIoU module accelerates the convergence of predicted boxes, enhances the regression accuracy of predicted boxes, and increases the F1-score to 95.9%, AP@0.5 to 98.1%, and AP@[0.5:0.95] to 76.5% compared to the original YOLOv5s.

**Table 1:** Results of the ablation experiments.

ID	EIoU	CBT	CBAM	AP@0.5(%)	AP@[0.5:0.95] (%)	F1(%)
1				97	69.5	92.5
2	✓			98.1	76.5	95.9
3		✓		97.8	75.8	95.8
4			✓	97.6	74.3	95.5
5	✓	✓		98	75.3	95.5
6		✓	✓	97.7	75.7	95.9
7	✓		✓	97.6	74.7	95.1
8	✓	✓	✓	98.2	77.1	96.2

The addition of CBT and CBAM modules improves the model’s ability to acquire global information and accurately capture the regions of interest. Performance is also enhanced compared to the baseline. These results show that the EIoU module, the CBT module, and the CBAM module are all effective in improving the accuracy of the detection model.

Then the YOLO-CFruit algorithm implemented by combining the sub-modules, compared with the original YOLOv5, AP@0.5 improved 1.2% relative to the first group, AP@[0.5:0.95] improved 7.6%, and F1-score improved 3.7%, which is higher than that of the sub-modules alone, which demonstrates that YOLO-CFruit performs very well in the detection of *Camellia oleifera* fruit in complex environments.

**Figure 7:**
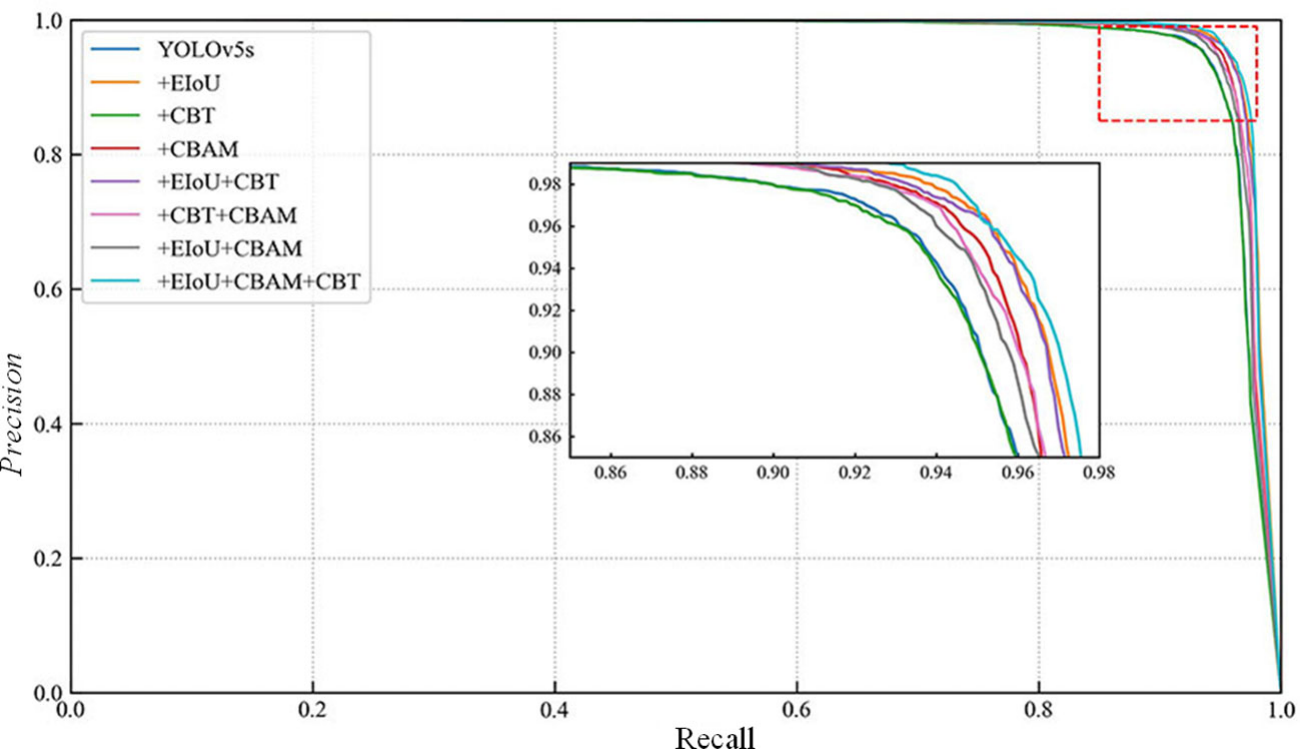
P-R curves for improved YOLOv5 model based on different model.

**Figure 7** shows the comparison of the P-R curves of the elimination of each sub-module in the ablation experiments, in which the P-R curve of YOLO-CFruit is closest to the upper right corner, which indicates that the better the model performance of YOLO-CFruit.

To qualitatively analyze the impact of CBAM, the Grad-CAM (Gradient-weighted Class Activation Mapping) technique (Selvaraju et al., 2017) was employed to compare different networks. Grad-CAM is a gradient-based visualization method that identifies the significance of spatial locations within convolutional layers, effectively highlighting regions of interest.

**Figure 8:**
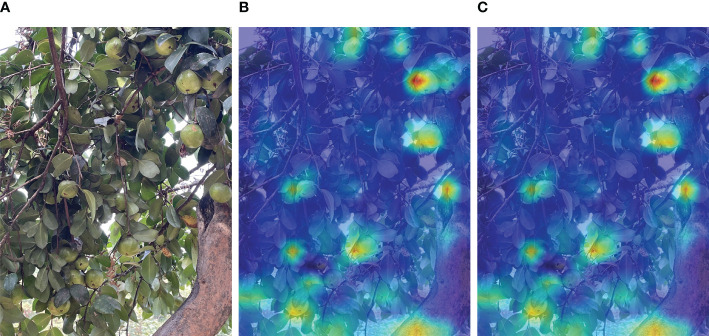
Grad-CAM visualization results. **(A)** original; **(B)** YOLOv5s; **(C)** CBAM.

**Figure 8** illustrates the Grad-CAM masks obtained from YOLOv5 combined with CBAM and YOLOv5 alone. The Grad-CAM masks of YOLOv5 with CBAM more accurately cover the regions of the target objects compared to YOLOv5 alone. This indicates that the combination of YOLOv5 with CBAM enables the network to effectively learn and consolidate features from the regions of interest, resulting in improved localization accuracy.

### 3.2 Performance of YOLO-CFruit model

**Figure 9:**
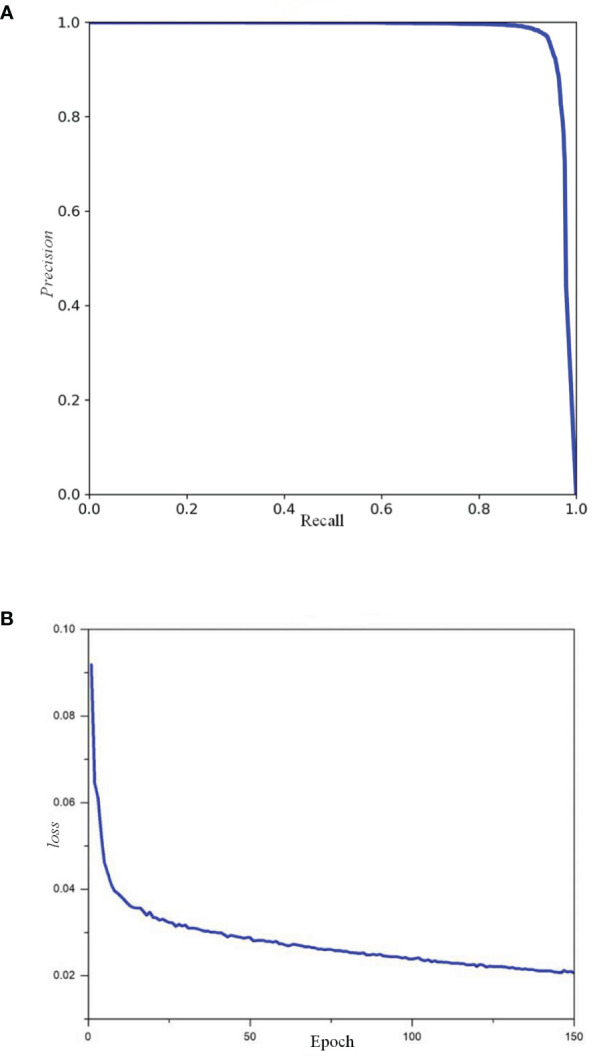
P-R curve and loss curve of YOLO-CFruit model. **(A)** P-R curve of YOLO-CFruit model; **(B)** loss curve of YOLO-CFruit model.

To evaluate the detection capabilities of the YOLO-CFruit model specifically for *Camellia oleifera* fruit, we applied YOLO-CFruit to the self-made *Camellia oleifera* fruit test set. The precision-recall curve and the loss during training curve is shown in **Figure 9**. The validation loss of YOLO-CFruit decreases from 0.091 to 0.023 during training. The precision (P), recall (R), average precision (AP), and F1 score of YOLO-CFruit are 98%, 94.5%, 98.2%, and 96.2%, respectively. Therefore the model can maintain a high detection performance in the detection of *Camellia oleifera* fruits.

To further assess the model’s robustness across various lighting angles, we handpicked 120 images from the test set, encompassing three distinct lighting environments: natural light, backlight, and exposure. These images were categorized into three groups based on these lighting conditions. Within each group, we tallied the total count of *Camellia oleifera* fruits, along with the number of missed detections and false detections. The statistical findings are presented in the **Table 2**. The results demonstrate that the YOLO-Cfruit model adeptly identifies the majority of oleander fruits across diverse lighting scenarios, exhibiting a low rate of false detections and missed detections.

**Table 2:** Detection results under different lighting conditions.

Scenario	Detected	Undetected	False	Undetection rate(%)	False detection rate (%)
natural light	172	12	1	6.5	0.54
backlight	276	26	2	8.6	0.66
exposure	270	34	4	11.2	1.3

**Figure 10:**
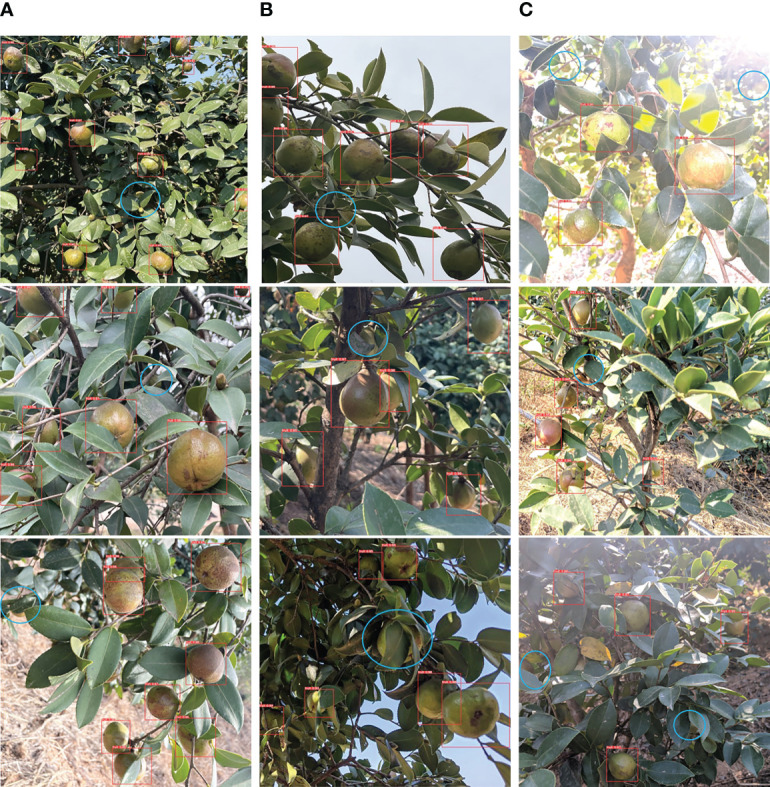
YOLO-CFruit detection results under different light environments. **(A)** natural light; **(B)** back light; **(C)** sidelight.

As shown in **Figure 10**, most of the *Camellia oleifera* fruits that were missed in the three different lighting scenarios were either heavily occluded or in an exposed environment. This is because there are few features that can be effectively extracted, so the probability of missed detection is relatively high.

When detecting *Camellia oleifera* fruit in practical application scenarios, the captured images often contain numerous fruits with varying sizes, severe occlusions, and disordered densities. These factors make it challenging to detect and leads to low detection accuracy. By YOLO-CFruit to effectively detect *Camellia oleifera* fruit, it can provide a feasible solution for deep neural networks in agriculture.

### 3.3 Comparison of results with other detection models

**Figure 11:**
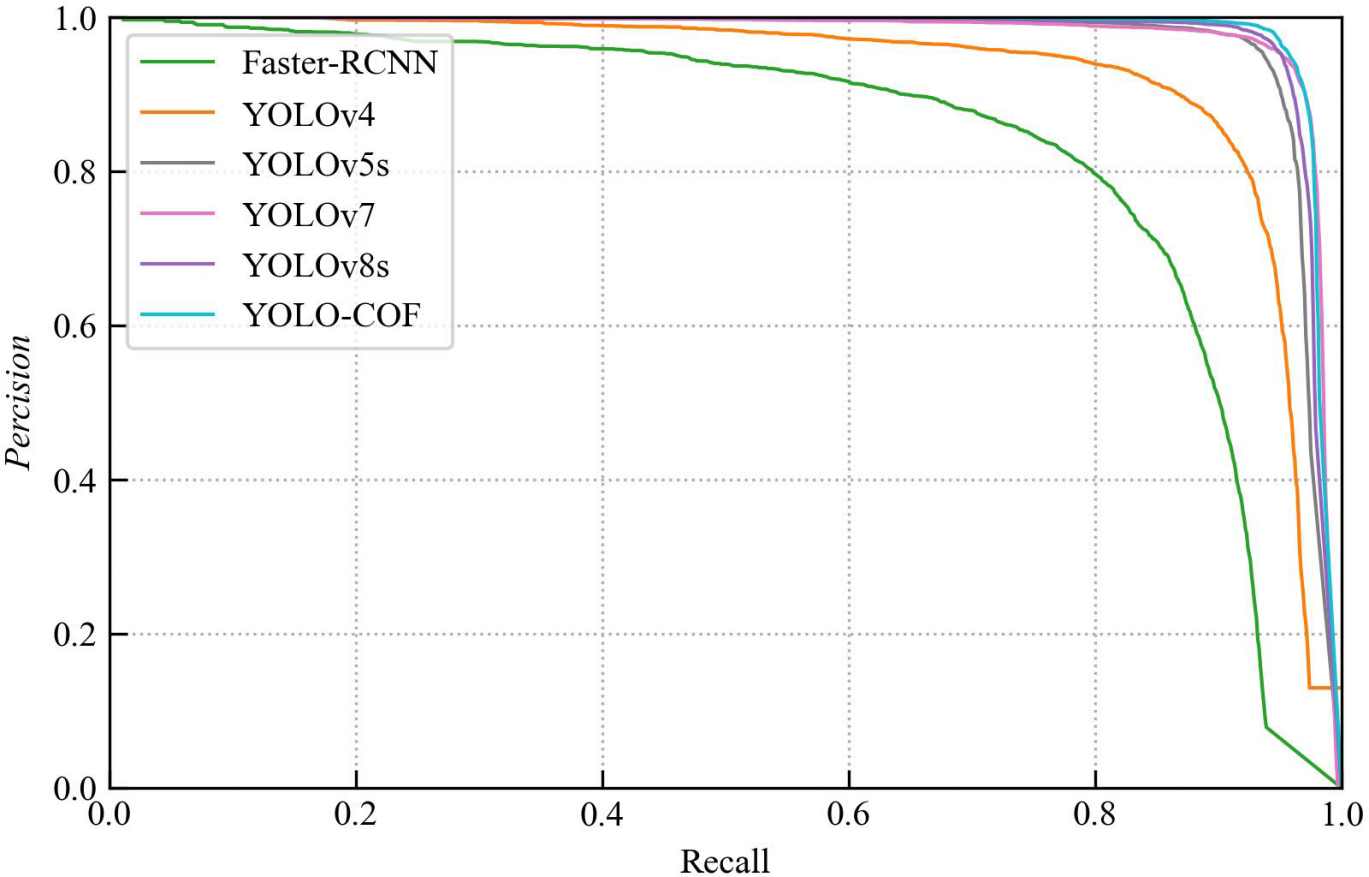
P-R curves for different detection models.

For a comprehensive assessment of YOLO-CFruit, we conducted a comparative assessment with contemporary models, including Faster-RCNN, YOLOv4, YOLOv7, YOLOv8s and the original YOLOv5s. Employing an identical test dataset and experimental conditions, we scrutinized their prediction outcomes. **Table 3** and **Figure 11** encapsulate the specific outcomes.

**Table 3:** Comparison of results with other detection models.

Model	AP(%)	F1(%)	FPS(%)	Weight(MB)
Faster-RCNN	82.9	79.9	7.6	382.18
YOLOv4	88.6	80.0	9.1	61.77
YOLOv5s	97	92.5	20.88	13.72
YOLOv7	97.8	94.7	9.16	71.32
YOLOv8s	97.6	94.2	9.04	21.46
YOLO-CFruit	98.2	96.2	19.02	11.77

As detailed in **Table 3**, among diverse detection metrics, YOLO-CFruit achieves a ap@0.5 reaches 98.2%, outperforming Faster-RCNN, YOLOv4, the original YOLOv5, YOLOv7 and YOLOv8s by 15.3%, 9.6%, 1.2%, 0.4% and 0.6%, respectively. The F1-score attains 96.2%, demonstrating superiority over Faster-RCNN, YOLOv4, the original YOLOv5, YOLOv7 and YOLOv8 by margins of 16.3%, 16.2%, 3.7%, 1.5% and 2.0%, correspondingly. Both of these pivotal metrics stand above those of the other models. The discernible trend portrayed in **Figure 11** highlights YOLO-CFruit’s P-R curve converging towards the upper right corner more closely compared to alternative models. Collectively, these findings conclusively affirm that YOLO-CFruit excels in terms of detection accuracy, surpassing its counterparts in the realm of target detection models.

As for the model size, the model size of YOLO-CFruit is 11.77 Mb, which is 370.41Mb, 50 Mb, 1.95 Mb, 9.69Mb, and 59.55 Mb smaller than Faster-RCNN, YOLOv4, the original YOLOv5s, YOLOv7, and YOLOv8s, respectively, indicating that YOLO-CFruit can be better adapted to the recognition system of the harvesting robots, which is conducive to model deployment and migration.

Although slightly lower than the original YOLOv5s in terms of inference speed, it performs well in all other detection metrics, so YOLO-CFruit can be used for *Camellia oleifera* fruit recognition in complex environments.

**Figure 12:**
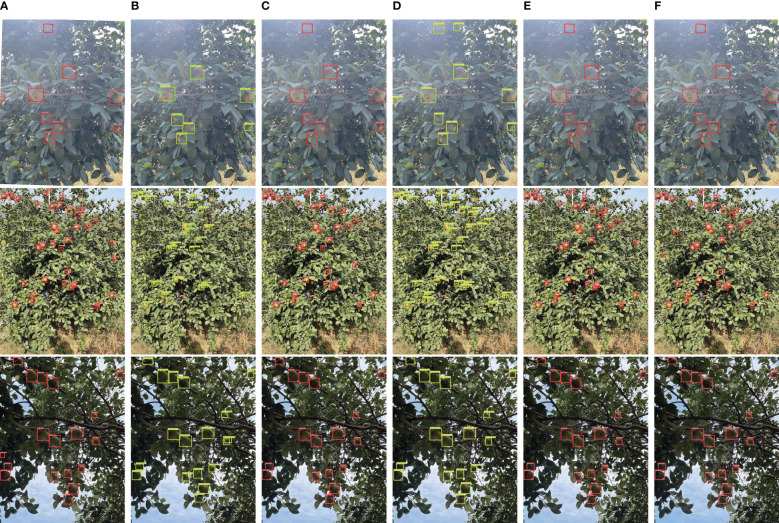
Comparison of YOLO-CFruit detection with four classical models under exposure conditions, natural light conditions, inverse conditions. **(A)** Faster-RCNN; **(B)** YOLOv4; **(C)** YOLOv5s; **(D)** YOLOv7; **(E)** YOLOv8s; **(F)** YOLO-CFruit.

**Figure 12** compares the detection results of YOLO-CFruit over other target detection algorithms in complex scenes under different lighting conditions. It can be seen that the YOLO-CFruit model can detect *Camellia oleifera* fruits missed by other models in different scenes. This shows that YOLO-CFruit can be more suitable for *Camellia oleifera* fruit detection than other models in complex scenes.

Overall, YOLO-CFruit can detect all *Camellia oleifera* fruit with the highest localization accuracy. It has enormous potential applications for collecting and detecting on mobile devices with limited computing capabilities.

Our improved YOLO-CFruit model achieves an impressive average precision(AP) of 98.2% with a FPS of 19.02, surpassing the performance of YOLOv4, YOLOv5S, YOLOv7, YOLOv8 models, and Faster-RCNN. This model shows significant potential for detecting and harvesting *Camellia oleifera* fruit using mobile devices with limited computational power. Implementing automatic harvesting based on this model could lead to cost reduction, improved efficiency, and benefit the *Camellia oleifera* fruit industry and local economy.

Cultivating *Camellia oleifera* fruit trees within an intricate and open environment presents inherent challenges to achieving accurate fruit detection. In response, we introduce YOLO-CFruit, a deep learning-based model meticulously designed for the purpose of detecting *Camellia oleifera* fruit.

However, we observed in our experiments that the model has a higher probability of missed detection and low precision localization when severe occlusion is present. Therefore, further optimization and improvement of the proposed method are necessary. In the future research we plan to collect more images using different methods and tools and build a larger dataset for detecting *Camellia oleifera* fruit. In particular, adding images of heavily occluded oleander fruits improves the ability to extract features of *Camellia oleifera* fruits in occluded situations. In addition, we aim to further optimize the network model to reduce the computational cost while maintaining high detection accuracy and improving the inference speed of YOLO-CFruit. And the model will be further applied to the detection of ripeness of *Camellia oleifera* fruit.

## 4 Conclusions

In this study, the proposed YOLO-Cfruit network model is used for the recognition of *Camellia oleifera* fruits in natural environments. The necessity for this research is driven by the critical need for accurate and efficient fruit detection to facilitate automated harvesting processes, which can substantially enhance productivity and reduce labor costs in the agricultural sector.

The YOLO-CFruit model has proven its effectiveness through rigorous evaluation, showcasing a mean average precision of 98.2%, a recall of 94.5%, precision of 98.0%, and an impressive F1 score of 0.962. These exemplary metrics, aligned with well-known objective evaluation standards, underscore the model’s high accuracy and reliability in fruit detection under diverse conditions.

Moreover, the model’s efficiency is evidenced by a FPS of 19.02, positioning YOLO-CFruit as a viable candidate for real-time applications. This swift performance, coupled with its superior accuracy, sets YOLO-CFruit apart from its counterparts, such as Faster-RCNN, YOLOv4, YOLOv5s, YOLOv7 and YOLOv8, in both average accuracy and overall performance.

In conclusion, the YOLO-CFruit model not only meets but exceeds the current benchmarks for object detection models, offering a compelling solution for the automated harvesting of *Camellia oleifera* fruits. The contributions of this study are multifaceted, including the development of a high-performing detection model and the potential to revolutionize agricultural practices. Future work will focus on further refining the model and exploring its applicability to other agricultural products, thereby expanding the impact of our research.

## Data Availability

The raw data supporting the conclusions of this article will be made available by the authors, without undue reservation.
